# To vaccinate or not to vaccinate? Dark triad personality traits in the context of decision about vaccination against COVID-19

**DOI:** 10.3389/fpsyt.2025.1582077

**Published:** 2025-08-07

**Authors:** Karina Badura-Brzoza, Patryk Główczyński, Paweł Dębski, Zenon Brzoza

**Affiliations:** ^1^ Clinical Department of Psychiatry, Medical Faculty in Zabrze, Medical University of Silesia in Katowice, Tarnowskie Góry, Poland; ^2^ Institute of Psychology, Department of Psychology, Humanitas University in Sosnowiec, Sosnowiec, Poland; ^3^ Clinical Department of Internal Medicine and Allergology, Endocrinology and Gastroenterology, Institute of Medical Sciences, Opole University, Opole, Poland

**Keywords:** COVID-19 pandemic, vaccinations, dark triad, stress, anxiety, machiavellianism, psychopathy, narcissism

## Abstract

**Introduction:**

The COVID-19 pandemic and the related restrictions have changed the lives of people around the world. Compliance with the recommendations issued by the WHO and the ministries of health of individual countries was important in order to limit the transmission of the SARS-COV-2 virus. Over time, public vaccinations against COVID-19 appeared, which gave rise to a wave of anti-vaccine demonstrations.

**Aim of the study:**

Bearing in mind the hypothesis about the relationship between the characteristics of the Dark Triad and pro-health behaviors, The aim of the study was to analyze the possible correlations between Dark Triad personality traits, the levels of anxiety and depressive symptoms and vaccination behavior.

**Material:**

419 people participated in the study, including 317 people aged 35.87 ± 13.65 SD years who received the Covid-19 vaccination and 102 people aged 38.18 ± 12.13 SD years who decided not to receive the vaccine.

**Methods:**

The Dirty Dozen Scale (DDS), the Perceived Stress Scale (PSS-10) and the State and Trait Anxiety Inventory (STAI-X2) were used in the study.

**Results:**

On the Dirty Dozen Scale, the vaccinated group had an average score of 23.29 ± 10.10 SD and the unvaccinated group had an average score of 20.81 ± 8.84 SD. Comparative analysis of the study groups showed statistically significant differences both in the overall DDS score and in its subscales - machiavellianism and narcissism. There were no differences in the psychopathy subscale. When analyzing the relationship between anxiety as a trait and stress assessed with the PSS-10 scale, no statistically significant correlations were found in the overall score on the DDS scale, as well as in the subscales of machiavellianism, psychopathy and narcissism.

**Conclusions:**

In the study, people with features of machiavellianism and narcissism more often decided to vaccinate against COVID-19, such a relationship could not be established for people with features of psychopathy. Aversive personality traits that are part of the Dark Triad (DD) are associated with non-compliance with the imposed norms or sanitary regime related to the pandemic, but they do not exclude concern for the health of the individual. Further research is needed on the Dark Triad in the context of social and cultural differences and in the field of undertaking pro-health behaviors.

## Introduction

The effectiveness and safety of the method of treatment, which are preventive vaccinations, is constantly questioned. This topic, although still present in groups of young parents, has become a popular topic in recent years due to the outbreak of the COVID-19 pandemic. Despite the fact that vaccinations are one of the pillars of modern medicine, in almost every year scientists observe the increase reluctance to this form of disease prevention. The problem of reluctance to prevent vaccinations has been known to mankind since Jenner’s invention, but it gained momentum in connection with the publication of an article in the Lancet journal about the link between the MMR vaccine and the etiology of autism spectrum disorders (1–4). The consequence of the activity of anti-vaccination movements is the increase in the incidence of diseases such as whooping cough and measles, currently observed in many countries (5, 6). The next wave of anti-vaccine attitudes emerged in connection with the COVID-19 pandemic. The outbreak of the pandemic, in the absence of an effective method of treatment, forced the governments of many countries to take measures to reduce the transmission of the virus (7). At the same time, scientists began researching an effective method of treatment, focusing their efforts on two aspects: the invention of a drug that would enable the fight against infections in already sick patients, and the invention of a vaccination that would prevent infection or make infections milder (8, 9). At the beginning of 2021, the first vaccination against the SARS-CoV-2 virus was introduced. The attitudes of some people towards this method of treatment were negative, pharmaceutical companies were accused of too short a research period for the new vaccine, many side effects were attributed to it, and some people expressed extreme views that it was a way to eliminate humanity. Protective vaccinations, as well as compliance with epidemic recommendations, are considered one of the most important factors in controlling the transmission of the pathogen. Nevertheless, many people accustomed to broad civil liberties opposed the restrictions introduced in this period, and later also vaccinations (10).

The reasons for such attitudes are varied. Some of them may be associated with specific personality traits that make these people non-conformist, have difficulty accepting scientific arguments, are suspicious of new treatment methods, or have problems accepting decisions made by people commonly regarded as authorities in a given field. Identification of psychological factors influencing making or abandoning certain decisions or behaviors during the COVID-19 pandemic seems to be an interesting area of research. So far, there have been few analyzes of socially undesirable personality traits such as psychopathy, narcissism and machiavellianism, which make up the Dark Triad, in the context of health-prophylactic behaviors (11, 12). The theme of the Dark Triad is relatively popular in the field of social research, in which individuals displaying these features are focused on egotistic activities to achieve personal success, striving to achieve only their own goals (13). These features also include unethical and antisocial behavior, impulsiveness and recklessness, limited compassion, alexithymia and low agreeableness (14). Some of these attitudes, including a narcissistic need to admire or a sense of self-importance, may be conducive to the implementation of pro-health behaviors - including vaccination against COVID-19. However, taking into account selfishness and anti-social behavior, these people could also have a significant difficulty in complying with pandemic restrictions and accepting new methods of treatment. To better understand the aim of the research, authors asked themselves questions: (1) Assessment of differences in the dark triad of personality and the intensity ofnstress and anxiety symptoms between people declaring vaccination against COVID-19 and those who did not get vaccinated. (2) Assessment of the relationships between the intensity of dark triad personality traits and the intensity of stress and anxiety symptoms in the group of people vaccinated against COVID-19, as well as in the group of people who were not vaccinated. (3) Assessment of the relationships between selected socio-demographic characteristics and the decision to get vaccinated against COVID-19.Whatsmore, authors put forward hypotheses: (1) Higher levels of narcissism will be positively associated with vaccination behavior due to increased self-focus and perceived invulnerability. (2) Higher levels of Machiavellianism and psychopathy will be negatively associated with vaccination behavior, reflecting distrust in authorities, low empathy, and risk-taking tendencies (3) Elevated anxiety symptoms will be associated with increased likelihood of vaccination, driven by health-related fear and a desire for safety. (4) Higher depressive symptoms will be associated with lower vaccination intent, potentially due to reduced motivation and pessimism about health outcomes. (5) The Dark Triad traits will moderate the relationship between affective symptoms and vaccination behavior.

## The aim of the study

The aim of the study was to analyze the possible correlations between Dark Triad personality traits, the levels of anxiety and depressive symptoms and vaccination behavior.

### Participants

The study was conducted in January-September 2022 in the Polish population. The research was designed in a cross-sectional model. The study was conducted online, using the convenience sampling method. Respondents were recruited via social media like Facebook. There were 419 people among the respondents, including 317 people aged 18 to 76, average age 35.87 ± 13.65 SD, who declared themselves vaccinated against Covid-19, and 102 people aged 18 to 75, average age 38.18 ± 12.13 SD years who have not received the COVID-19 vaccine. All respondents agreed to participate in the project. The sociodemographic characteristics of the respondents are presented in **Table 1**.

**Table 1:** Socio-demographic characteristics of the studied groups.

	VACCINATED	NOT-VACCINATED
	n = 317	n = 102
EDUCATION LEVEL		
HIGHER EDUCATION	>196	>48
HIGH SCHOOL/STUDENTS	>107	>47
MIDDLE SCHOOL	>5	>1
TECHNICAL SECONDARY SCHOOL	>9	>6
EMPLOYMENT		
FULL-TIME WORK	>180	>61
SELF-EMPLOYED	>35	>17
PART-TIME WORK	>24	>5
STUDENT	>50	>4
RETIREMENT	>12	>0
UNEMPLOYED	>16	>7
RELATIONSHIP STATUS		
SINGLE	>85	>22
IN RELATIONSHIP	>232	>80
HAVING CHILDREN		
YES	>148	>65
NO	>169	>37
DOMICILE		
VILLAGE	>49	>25
CITY	>268	>77
SHOULD PEOPLE VACCINATE AGAINST COVID-19?		
YES	>278	>11
NO	>8	>36
I DON’T KNOW	>31	>55
HAVING CHRONIC SOMATIC DISEASES		
YES	>66	>21
NO	>251	>81
SMOKING CIGARETTES		
YES	>51	>30
NO	>266	>72
QUARANTINE HISTORY		
YES	>162	>46
NO	>155	>56
SHOULD PEOPLE VACCINATE AGAINST (OTHER THAN COVID-19) DISEASES?		
YES	>303	>85
NO	>14	>17
DID YOU HAVE COVID-19 (POSITIVE PCR TEST)?		
YES	>115	>38
NO	>202	>64
DID SOMEONE FROM YOUR FAMILY HAVE COVID-19?		
YES	>263	>83
NO	>54	>19
DID YOU VACCINATE IMMEDIATELY, WHEN YOU COULD DO IT?		
YES	>257	
NO, LATER	>60	

### Methods

The following psychometric questionnaires were used to assess the examined parameters:

1. Original demographic data questionnaire.
2. The Dirty Dozen Scale, in the Polish adaptation by Czarna et al., is a tool used to measure the severity of antisocial personality traits. This questionnaire is used to assess three components of the so-called The dark triad of personality - subclinical psychopathy, subclinical narcissism and machiavellianism. The tool consists of twelve statements relating to socially undesirable tendencies in human behavior. The respondent has the opportunity to respond to them on a 5-point Likert scale, where 1 means “not true at all” and 5 “extremely true”. The questionnaire makes it possible to assess the overall severity of antisocial tendencies (the Dark Triad), as well as to obtain results in three subscales - psychopathy, narcissism and machiavellianism. There are 4 test items for each subscale. The tool has satisfactory psychometric properties. The internal consistencies of the scales were as follows: α = 0.64 for psychopathy, α = 0.81 for narcissism, α = 0.83 for Machiavellianism. The factor structure was also examined and the criterion validity of the tool was confirmed (15).
3. The Perceived Stress Scale (PSS-10) by Cohen, Kamarck and Mermelstein, in the Polish adaptation by Juczyński and Ogińska-Bulik, is a tool used to measure the intensity of stress related to one’s own life situation. The rating on this scale applies to the past month. Stress is understood here as a reaction to difficult experiences. This tool consists of 10 items, to which the respondent refers on a five-point scale, where 0 means “never” and 4 “very often”. On average, the test takes 5 minutes to complete. The score on the scale ranges from 0 to 40. The higher the score, the greater the level of stress. The result is obtained by summing up the digits marked by the examined person in individual positions, while some of the results should be reversed in accordance with the rule 0 = 4, 1 = 3. The PSS-10 tool is a standardized and normalized tool in Polish cultural conditions. Sten standards ranging from 1 to 10 are used. Normalization data are included in the official manual presenting adaptation process. The tool has satisfactory psychometric properties in terms of validity and reliability. Cronbach’s alpha coefficient for the whole tool was 0.86 (16).
4. State-Trait Anxiety Inventory (STAI), subscale X-2, by Spielberger et al., Polish study by Spielberger, Strelau, Tysarczyk and Wrześniewski. The inventory contains 40 statements, half of which assess anxiety as a relatively constant personality trait (X2), and the remaining anxiety as a situationally conditioned state (X1). The obtained results can be normalized using the sten scale. The study used only the subscale assessing anxiety as a trait (X2). The internal consistencies of the scale were for adult people: aged 21–40 years old - α = 0.85, aged 41-54 - α = 0.90, aged 55-69 - α = 0.82, and aged 70-79 - α = 0.84 (17).

### Statistical analysis

Standard statistical procedures were used in the analyses. Normality of the distributions of variables for each of the studied groups was assessed separately based on the results of the Shapiro-Wilk test. In the case of all variables, their distributions deviated from the normal distribution, therefore it was decided to use nonparametric statistics in further analyses. The Mann-Whitney U test was used to assess the significance of differences between the study groups. Spearman’s rank correlation coefficient was used to assess the relationships between the data. The associations of dichotomized socio-demographic variables with COVID-19 vaccination were tested using the chi-square test. The significance level of p<0.05 was assumed as statistically significant. The calculations were made in the Statistica program (Statistica.INC 13.3).

### Ethics of conducted research

The Bioethics Committee of the Medical University of Silesia approved the study (PCN/0052/KB1/45/22). The participants were informed about the anonymity and confidentiality of the research. Moreover, they were informed that they could stop the study at any time. Information about the study and informed consent was included in the first part of the prepared form.

## Results

The study involved 419 people, including 317 people who were vaccinated (VG) (209 women, 108 men) and 102 people were unvaccinated (UVG) (70 women and 32 men).

1. The Dirty Dozen Scale (DDS)

In the DDS for vaccinated persons, the mean score was 23.29 ± 10.10 SD points and the unvaccinated group had an average score of 20.81 ± 8.84 SD points. Comparative analysis of the study groups showed statistically significant differences both in the general DDS scale and in its subscales - machiavellianism (MACH) and narcissism (NAR). No differences were found in the psychopathy (PSYCH) subscale (**Table 2**).

**Table 2:** Differences between vaccinated and unvaccinated groups.

	Vaccinated (n=317)				Unvaccinated (n=102)				U Mann-Whitney	
	Mean	SD	Median	Shapiro-Wilk (p)	Mean	SD	Median	Shapiro-Wilk (p)	Z	P
**DD**	23.292	10.105	23.000	**0.000**	20.807	8.845	21.000	**0.000**	2.523	0.006*
**MACH**	6.976	3.841	6.000	**0.000**	5.817	3.046	5.000	**0.000**	3.125	0.001*
**NAR**	9.074	4.614	9.000	**0.000**	7.889	4.124	8.000	**0.000**	2.409	0.009*
**PSYCH**	7.243	3.427	7.000	**0.000**	7.101	3.389	7.000	**0.000**	0.492	0.610
**PSS-10**	19.772	6.656	20.000	**0.000**	18.724	7.314	20.000	**0.009**	1.100	0.258
**STAI X2**	46.991	7.682	46.000	**0.000**	45.889	7.966	45.000	**0.032**	1.280	0.327

DD, Dark Triad total score; MACH, Machiavellianism; NAR, narcissism; PSYCH, psychopathy; PSS-10, perceived stress; STAI X2, The State-Trait Anxiety Inventory; Shapiro-Wilk (p), assessment of the normality of the variable distribution using the Shapito-Wilk test, * statistically significant with p<0.05. The bold values mean statistically significant with p<0.05.

2. Perceived Stress

Analyzing the results obtained in the PSS-10 questionnaire, the average score of 19.77 ± 6.65 SDpoints was obtained in the group of vaccinated persons, and in the group of unvaccinated persons the average score was 18.72 ± 7.31 SD points, which, when converted into sten values, allows us to define the level of stress as high. Comparing the two groups, no statistically significant differences were found (**Table 2**).

3. Anxiety tendencies

In the group of vaccinated persons, the average score in the questionnaire STAI examining anxiety as a trait (X2) was 46.99 ± 7.69 SD points, and in the group of unvaccinated persons the result was 45.88 ± 7.96 SD points. (**Table 1**). There were no statistically significant differences between the study groups (**Table 2**).

4. Analysis of the relationship between the studied factors

In both study groups, there was no relationship between the results obtained in DDS and in the subscales (machiavellianism, narcissism and psychopathy) with the results obtained in the PSS-10 stress scale and with the results obtained in the anxiety as a personality trait scale (STAI-X2) (**Tables 3**, **4**).

**Table 3:** Correlations between the variables in the vaccinated group.

n=317	DD	MACH	NAR	PSYCH	PSS-10	STAI X2
DD	1.000	0.837*	0.826*	0.676*	0.0861	0.084
MACH		1.000	0.598*	0.498*	0.073	0.079
NAR			1.000	0.351*	0.045	0.147
PSYCH				1.000	0.036	0.030
PSS-10					1.000	0.656*
STAI X2						1.000

PSS-10, perceived stress; STAI X2, trait anxiety; DD, Dark Triad total score; MACH, Machiavellianism; NAR, narcissism; PSYCH, psychopathy; * statistically significant with p<0.05. The bold values mean statistically significant with p<0.05.

**Table 4:** Correlations between the variables in the unvaccinated group.

n=102	DD	MACH	NAR	PSYCH	PSS-10	STAI X2
DD	1.000	0.772*	0.854*	0.705*	-0.08	0.093
MACH		1.000	0.601*	0.436*	-0.041	-0.011
NAR			1.000	0.352*	0.084	0.143
PSYCH				1.000	-0.105	0.034
PSS-10					1.000	0.621*
STAI X2						1.000

PSS-10, perceived stress; STAI X2, trait anxiety; DD, Dark Triad total score; MACH, Machiavellianism; NAR, narcissism; PSYCH, psychopathy; * statistically significant with p<0.05. The bold values mean statistically significant with p<0.05.

4. The associations of dichotomized socio-demographic variables with COVID-19 vaccination

Socio-demographic variables were dichotomized in terms of education (one group was created from people with higher education and students, the other from people with other education levels) and employment (one group was created from people who were employed, and the other group from people who were not). The remaining socio-demographic variables were initially dichotomized (**Table 1**). An association with the decision to vaccinate was demonstrated for education, place of residence, having children, smoking. People with higher education (and students) (chi2 = 35.460; p < 0.001), city dwellers (chi2 = 4.349; p = 0.037), people without children (chi2 = 8.963; p = 0.003), and non-smokers (chi2 = 8.784; p = 0.037) were significantly more often vaccinated against COVID-19.

## Discussion

In order to properly analyze the relationship between the Dark Triad and pro-health behaviors in terms of vaccination, it is necessary to analyze its components - machiavellianism, psychopathy and narcissism (18). Each of the domains of the Dark Triad is a complex set of different traits, but common to all of them is the anti-social attitude and behavior of the individual, as well as the difficulties he exhibits in respecting social norms (13). The period of the COVID-19 pandemic was a time when the rules of social functioning gained special importance and behavior was modified to a large extent by new factors. Modyfying to specific conditions required adopting specific attitudes, which could be a significant challenge for people with Dark Triad personality traits (19). One of the attitudes was to follow the restrictions - keeping social distance, complying with the rules of wearing masks indoors or outside houses and the other was to decide to get vaccinated. Both attitudes have both a social and an individual context. Whatsmore, both may be related to building a social image, and may result from internal motivation. Compliance with the rules imposed by the governments of countries in the face of a wave of increasing COVID-19 cases was defined as one of the most important factors modifying the course of the pandemic. Some studies have shown that people with Dark Triad traits resisted the restrictions (20). Traits such as extroversion and openness are usually associated with narcissism and psychopathy. Earlier research by Clark and Zajenkowski showed that extroverted people have more difficulties with observing the rules of social distancing than introverted people (21, 22), although not all studies confirm this (23). So far, the phenomenon of the Dark Triad personality traits (DT) has been studied in the context of undertaking risky behaviors by these people, both socially and healthily (24, 25). Chavez-Ventura pointed out that the amoral behavior characteristic of people with DT favors uncritical opposition to wearing masks in public places and compliance with any recommendations based on social obedience (26). Ahzadeh et al. tried to explain this opposition to the pandemic rules by obtaining a high score in terms of belief in conspiracy theories in people with DT, in all three personality domains, but most pronounced in people showing features of narcissism and machiavellianism (27). Similar conclusions regarding narcissism were drawn by Cichocka, who cited the need for control and the belief in one’s uniqueness as the reason for such behavior (28). Another study that showed the relationship between narcissism and pro-health behavior was the analysis of Dębska et al. (29). Therefore, it can be hypothesized that people with Dark Triad traits may be more inclined primarily due to selfish motives to care only for their own health. Our study showed that vaccinated individuals scored higher on The Dirty Dozen Scale, with statistical significance in machiavellianism and narcissism. This could confirm the supposition that such features as egoism, the need for superiority or a tendency to manipulate may make these individuals more willing to decide to get vaccinated against COVID-19 solely for reasons of taking care of their own interests. The British study of Hughes and Machan which was carried out before the introduction of the COVID-19 vaccine, analyzed attitudes towards this form of disease prevention. The results were unequivocal, all people with DT features were skeptical about the sense of developing a vaccine and its safety (30). This fact sheds a slightly different light on the perception of the idea of ​​preventive vaccination, in which the argument of the common good is most often raised. It turns out, therefore, that perhaps it is selfishness that is the main reason for the decision to vaccinate, and not altruism. An attempt to explain this phenomenon can be made by the words of the Polish philosopher and ethicist Władysław Tatarkiewicz, who recognized altruism as the subtlest version of egoism (31).

Howard came to slightly different conclusions. In his study from 2022, machiavellianism had no influence on the decision (positive or negative) to vaccinate, while narcissism was associated with a greater tendency to undertake health-risk behaviors and refusal to be vaccinated (32). Whatsmore, Danish researchers Hatemi and Fazekas came to very interesting conclusions, dividing narcissism into “grandiose” and “vulnerable”, which significantly differentiated attitudes towards vaccination. Persons with features of grandiose narcissism showed oppositional behavior towards pandemic restrictions, while persons with features of vulnerable narcissism, more sensitive to social judgments, more often decided to be vaccinated (33). Another feature differentiating the groups in our study was machiavellianism, with a higher score on this scale obtained by vaccinated persons. Perhaps people with these personality traits have found that being vaccinated could be helpful in achieving some of their important life goals. Being vaccinated against COVID-19 during the pandemic significantly facilitated the implementation of plans, e.g. going abroad, getting a job or freely visiting gastronomic establishments (34, 35). Contrary to the two previous components, no statistical significance of the difference between the study groups in the psychopathy domain was found. Howard emphasized that psychopathy is rather related to the refusal of vaccinations and health-risk behaviors (32), which was also confirmed by Dębska (29). It seems that people with such characteristics do not attach importance to either social rules or the protection of their own health. Other variables analyzed in our study were anxiety as a personality trait and symptoms of stress. It seems that both of these factors may influence vaccination decisions - both positively and negatively (36, 37). However, in our study, the level of personality anxiety did not statistically differentiate the study groups, nor did the level of stress assessed with the PSS-10 scale. There was also no statistically significant relationship between the above-mentioned factors and the results obtained in the Dark Triad scale. No studies on this topic have been found either. In our study, people with higher education (and students), city dwellers, people without children, non-smokers and respondents who believe that people should be vaccinated against infectious diseases were significantly more often vaccinated against COVID-19. Analyzing these aspects one by one - higher education as a factor favoring the decision to vaccinate should not seem surprising, but this relationship is not at all obvious. While such relationships were observed in Humer et al. or Scharff et al. (38, 39), Trepanowski approached it in a different way, analyzing the regression - higher education led to lower vaccination rates, especially in economically developed countries. It is worth noting that higher education does not have to include knowledge about vaccinations (40). City dwellers in our study were more willing to get vaccinated. In terms of residence and willingness to get vaccinated, Datta et al. reached similar conclusions (41), while Wu et al. reached completely different ones (42). It seems that the cultural context itself and trust in the government are important factors. In our analysis, non-smokers were more willing to make decisions about vaccination, which seems to be established in other studies (43, 44). Summing up, it can be said that socially aversive traits in the context of attitudes towards protecting one’s own health and that of others are a complex topic and require further research.

## Study limitations

The sample size is not large and limited to one population, which may affect representativeness. A relatively small size of the unvaccinated group could limit the generalizability of the findings. The Dirty Dozen test does not allow for point classification of subtypes of narcissism, Machiavellianism and psychopathy, the classification of which could be helpful in determining the relationship between them and the attitude towards COVID-19 vaccination. Another limitation related to the use of this tool is the low internal consistency of one of the scales - in the case of psychopathy it was α = 0.64. therefore, results obtained using this scale should be treated with caution. More comprehensive instruments, such as the Short Dark Triad (SD3) or the Narcissistic Personality Inventory (NPI) might have yielded more detailed and differentiated insights. While correlations between Dark Triad traits and vaccination behavior are identified, the directionality and underlying mechanisms remain unclear. Whatsmore, further exploration of potential moderating or mediating factors is warranted to clarify the outcome. A longitundital design would provide stronger evidence of causality. The study does not account for potential cultural or socio-political factors that could influence both personality expression and vaccination behavior. Authors are aware of the influence of that factors on decision about vaccinations.

## Conclusions

1. The surveyed people showing features of narcissism and machiavellianism more often made a positive decision regarding vaccination against COVID-19.

2. No relationship was found between psychopathic features and a positive or negative decision to receive a COVID-19 vaccination.

3. Anxiety as a personality trait and stress did not correlate with the results of the Dark Triad of Personality in both study groups.

4. Aversive personality traits that may predispose to non-compliance with social norms and the sanitary regime, however, do not preclude the individual from caring for his own health and undertaking pro-health behaviors beneficial from his individual point of view.

## Data Availability

The original contributions presented in the study are included in the article/supplementary material. Further inquiries can be directed to the corresponding author/s.
